# Association between meibomian gland dysfunction and compliance of topical prostaglandin analogs in patients with normal tension glaucoma

**DOI:** 10.1371/journal.pone.0191398

**Published:** 2018-01-31

**Authors:** Tae Hee Lee, Mi Sun Sung, Hwan Heo, Sang Woo Park

**Affiliations:** Department of Ophthalmology, Chonnam National University Medical School and Hospital, Gwangju, South Korea; Ophthalmology Clinic, University G. d’Annunzio of Chieti-Pescara, ITALY

## Abstract

**Purpose:**

The aim of this study was to investigate the association between tear film and meibomian gland parameters in patients with normal tension glaucoma (NTG), who underwent topical prostaglandin analog (PGA) monotherapy, and medication compliance.

**Methods:**

Ocular surface disease index (OSDI), Schirmer’s test, tear film break-up time (TBUT), keratoepitheliopathy (KEP) score with fluorescein, and meibomian gland parameters were assessed in 45 eyes of 45 patients with NTG (NTG group), who received topical PGA monotherapy for more than 1 year. The results were compared to those of 40 eyes of 40 normal subjects (control group). Medication compliance was assessed by an 8-item Morisky Medication Adherence Scale (MMAS-8). Multiple logistic regression analysis was used to identify the factors associated with medication compliance.

**Results:**

There was a significant difference in OSDI (P = 0.043), Schirmer’s test (P < 0.001), TBUT (P < 0.001), KEP score (P = 0.015) and all meibomian gland parameters (all P < 0.001) between two groups. When the NTG group was divided into compliant and non-compliant groups based on the scores of MMAS-8, 30 (75%) patients were classified into the compliant group. Multiple logistic regression analysis revealed that the lid margin score (OR, 0.256; 95% CI, 0.072–0.908, P = 0.035), meibum score (OR, 0.144; 95% CI, 0.023–0.915, P = 0.04), and meibo score (OR, 0.344; 95% CI, 0.140–0.845, P = 0.02) were significant factors associated with compliance in patients with NTG. The meibomian gland parameters showed a negative correlation with medication compliance (all P < 0.005).

**Conclusions:**

Malfunction of the meibomian glands can be an important clinical finding associated with compliance of PGA monotherapy in patients with NTG.

## Introduction

The manifestation of ocular surface discomfort is a frequent finding in patients with glaucoma treated with topical anti-glaucoma drugs. Ocular surface disease (OSD) has an overall prevalence of 42% (range 20–59%) in patients with glaucoma and is classified as severe in 36% of the patients (range 14–66%) [1]. The long term use of topical anti-glaucoma drugs may induce modifications of the ocular surface tissues and the adnexa, such as the conjunctiva, cornea, eyelids, periocular skin and meibomian glands [2–5]. These changes may be caused by the active ingredient, as well as the preservatives used in commercial medications. However, the mechanisms involved and the respective roles of the active compounds and preservatives in inducing the possible allergic, toxic, or proinflammatory effects of ophthalmic solutions are still being debated [6–8].

Patients with normal tension glaucoma (NTG) are usually treated with topical drugs to control intraocular pressure (IOP). Among currently available ocular hypotensive agents, prostaglandin analogs (PGAs) are the first-line choice for the treatment of NTG, because of their strong IOP-lowering effect, fewer systemic side effects, and need for less frequent dosing [9]. Recently, preservative free PGAs have been widely used. However, PGA preparations have traditionally been preserved with benzalkonium chloride (BAK), which causes damage to ocular tissue by inducing apoptosis and increasing the concentrations of inflammatory markers [10–12]. PGAs may also potentially be involved in meibomian gland dysfunction (MGD), an inflammation-driven eyelid disorder. Arita et al. reported that long-term use of anti-glaucoma drugs was associated with alterations in meibomian gland morphology and function [13]. Mocan et al. also demonstrated that long-term administration of PGAs was associated with an obstructive type of MGD [14]. These various damages caused by PGAs can have a negative impact on treatment compliance [15].

Ocular surface changes due to the use of anti-glaucoma drugs have been previously reported but have never been studied for factors associated with medication compliance.

The aim of this study was to investigate the association between the tear film and meibomian gland parameters and compliance of PGA monotherapy in patients with NTG and to analyze the factors associated with medication compliance.

## Methods

### Subjects

This cross-sectional study included patients with NTG treated with preserved topical PGAs and control subjects without any topical medications. The study protocol and informed consent were approved by the Chonnam National University Hospital Review Board. The study adhered to the tenets of the Declaration of Helsinki. The participants were informed about the study objectives and signed informed consent was obtained from all participants.

NTG was diagnosed based on the following criteria: glaucomatous optic neuropathy and a reproducible visual field (VF) defect, determined using a Humphrey Field Analyzer (Carl Zeiss Meditec Inc., Dublin, CA, USA) with the central 30–2 threshold test using SITA-standard test strategy; the moment of diagnosis the mean untreated IOP lower than 21 mmHg measured by Goldmann applanation tonometry; and a normal open angle on gonioscopy. Inclusion criteria were patients with NTG (NTG group), treated with either latanoprost (Xalatan^®^, Pfizer Inc., New York, USA), tafluprost (Taflotan^®^, Santen Pharmaceutical Co, Ltd, Osaka, Japan), or bimatoprost 0.01% (Lumigan^®^, Allergan Inc., Irvine, CA, USA) monotherapy, for more than 1 year. The BAK concentrations of latanoprost, tafluprost, and bimatoprost 0.01% were 0.02%, 0.001%, and 0.02%, respectively. The exclusion criteria were as follows: severe ocular trauma at any time, previous history of intraocular surgery or argon laser trabeculoplasty, intracranial lesion or neurologic disorder, current use of contact lenses, central corneal thickness <500 μm or >600 μm, presence of eyelid or eyelash deformity, history of recent ocular inflammation or infection, previous or current use of other ocular medications including artificial tear therapy, systemic treatments that are known to affect tear secretion, autoimmune disease, and any history or slit-lamp evidence of eye surface disorders. Subjects for normal control (control group) were recruited from those who came for a routine eye examination, patient relatives and hospital staff. The control group was matched by age and sex. The inclusion criteria for the normal controls were healthy subjects with no family history of glaucoma, no previous intraocular surgery, IOP ≤ 21mmHg, non-glaucomatous ONH and normal VF. The control group did not have clinical signs and/or symptoms of dry eye (Ocular surface disease index [OSDI] score <10) or significant ocular surface disease. The eight-item Morisky Medication Adherence Scale (MMAS-8) is one of the most widely used methods to assess patient adherence [16–19]. The MMAS-8 used in this study. The glaucoma medication compliance in the NTG group was evaluated using the MMAS-8. The total score of all items was calculated, ranging from 0 to 8, for adherence. The MMAS scores were characterized previously into the following three levels of adherence: high adherence (score, 8), medium adherence (score, 6 to < 8), and low adherence (score, < 6) [20].

### Clinical assessment of the ocular surface

The subjective symptoms were graded using the OSDI score (0 to 100), with higher scores representing greater disability [21]. The tear film break-up time (TBUT) and Schirmer’s test were performed as previously described [22,23]. Briefly, 2 μl of 1% fluorescein solution was instilled on to the inferior palpebral conjunctiva. The interval between the last blink and the appearance of the first precorneal hypofluorescent spot, streak, or other irregularity interrupting the normal homogenous fluorescein pattern was recorded as the TBUT (seconds) [22]. The Schirmer’s test was performed by instilling one drop of proparacaine 0.5% anesthetic, then waiting for 5 min. A standard Schirmer’s test strip was then placed in the lateral canthus for another 5 min, with the eye closed. The length of wetting of the strip was measured using the millimeter scale [23]. Keratoepitheliopathy (KEP) was scored by multiplying the area score (0–3) by the density score (0–3), after staining with 1% fluorescein dye [24].

### Evaluation of meibomian gland dysfunction

The following three parameters are the most commonly used methods to evaluate the morphological characteristics and function of the meibomian glands in clinical practice: abnormalities of the lid margins, expression of meibum, and gland dropout degree visualized by meibography [25]. Lid margin abnormalities were recorded according to the presence of the following four signs [26]: irregular lid margin, vascular engorgement, glandular orifice obstruction, and anterior or posterior displacement of the mucocutaneous junction. The eye was scored was from 0 to 4. To assess the expression of meibum semiquantitatively, the center of the upper tarsus was expressed using a thumb, and the meibum score was graded as follows [27]: grade 0 (clear meibum expressed easily), grade 1 (cloudy meibum expressed gently), grade 2 (cloudy meibum expressed with more than moderate pressure), and grade 3 (no meibum expressed even with hard pressure). Meibomian gland morphology was observed by Keratograph 5 M (OCULUS, Wetzlar, Germany), a noncontact, Placido ring- based corneal topographer [28,29]. Images of the meibomian gland were captured after eyelid eversion. The meibomian gland dropout degree was graded for each eyelid as the meibo score according to the following scale [26]: grade 0 (no loss of meibomian glands), grade 1 (loss of < 33% of the entire glands area), grade 2 (loss of area between 33% and 67%), and grade 3 (loss of > 67% of the entire area). The meibo score of each eye was calculated as the sum of the scores from the upper and lower eyelids, with a total meibo score per eye in the range of 0–6. In this study, the meibo score per eye was evaluated for each group and comparisons were made.

### Statistical analyses

SPSS version 18.0 (SPSS Institute Inc., Chicago, IL, USA) was used for the statistical analyses. The data were described as the mean (±SD). The normality of distribution was verified using the Shapiro-Wilk normality test. Differences in the various parameters between the two groups were evaluated using the chi-square test and the independent t-test. Multiple logistic regression analysis was used to evaluate the risk factors associated with glaucoma medication compliance. Each variable was first analyzed using a univariate model; all variables with a significant level (P < 0.10) were then evaluated using the multivariate model. The relationship between medication compliance and significant parameters was additionally examined using scatter plots and linear regression. The coefficient of determination (R^2^) in the linear regression was reported and statistical significance was considered as P < 0.05.

## Results

Overall, 85 subjects were included in this study, with 45 eyes in the NTG group and 40 eyes in the control group. Characteristics of the included subjects are presented in Table 1. The mean subject age was 60.11 ± 15.24 years and 59.58 ± 11.98 years in the NTG group and control group, respectively. There were no significant differences in age, gender, and IOP between the two groups. However, OSDI (P = 0.043), TBUT (P < 0.001), Schirmer’s test (P < 0.001), and KEP score (P = 0.015) had significant differences between the two groups. In addition, all meibomian gland parameters had significant differences between the groups (P < 0.001 for all parameters). The mean period of topical PGA administration in the NTG group was 42.93 ± 34.28 months.

**Table 1:** Demographics and clinical characteristics of subjects.

Variables	NTG group (n = 45)	Control group (n = 40)	P-value
**Age (yrs)**	60.11 ± 15.24	59.58 ± 11.98	0.859
**Sex (male/female)**	25/20	22/18	0.959
**IOP (mmHg)**	14.22 ± 2.06	14.68 ± 2.74	0.397
**MD (dB)**	-7.78 ± 7.27	-0.14 ± 1.20	< 0.001
**PSD (dB)**	6.70 ± 4.20	1.38 ± 0.27	< 0.001
**CCT (μm)**	543.19 ± 37.24	552.86 ± 32.71	0.627
**OSDI**	11.39 ± 5.52	8.96 ± 5.35	0.043
**TBUT**	4.36 ± 1.58	7.54 ± 2.98	<0.001
**Schirmer’ test**	6.44 ± 1.77	10.45 ± 5.51	<0.001
**KEP**	0.93 ± 1.23	0.40 ± 0.59	0.015
**Lid margin score**	1.53 ± 0.99	0.73 ± 0.88	<0.001
**Meibum score**	1.36 ± 0.80	0.33 ± 0.47	<0.001
**Meibo score**	2.49 ± 1.39	1.45 ± 1.01	<0.001
**Duration of therapy (months)**	42.93 ± 34.28	-	-
**Anti-glaucoma drugs (Latanoprost/Tafluprost/Bimatoprost)**	22/14/9	-	-

Data are expressed as mean ± standard deviation unless otherwise indicated.

NTG = normal tension glaucoma; IOP = intraocular pressure; MD = mean deviation; PSD = pattern standard deviation; CCT = central corneal thickness; OSDI = ocular surface disease index; TBUT = tear film break-up time; KEP = keratoepitheliopathy

The average score of the MMAS-8 was 5.82 ± 2.03 in the NTG group. We defined high and medium adherence as the compliant group and low adherence as the non-compliant group. The number of patients in the compliant group was 30 (75%).

**Fig 1:**
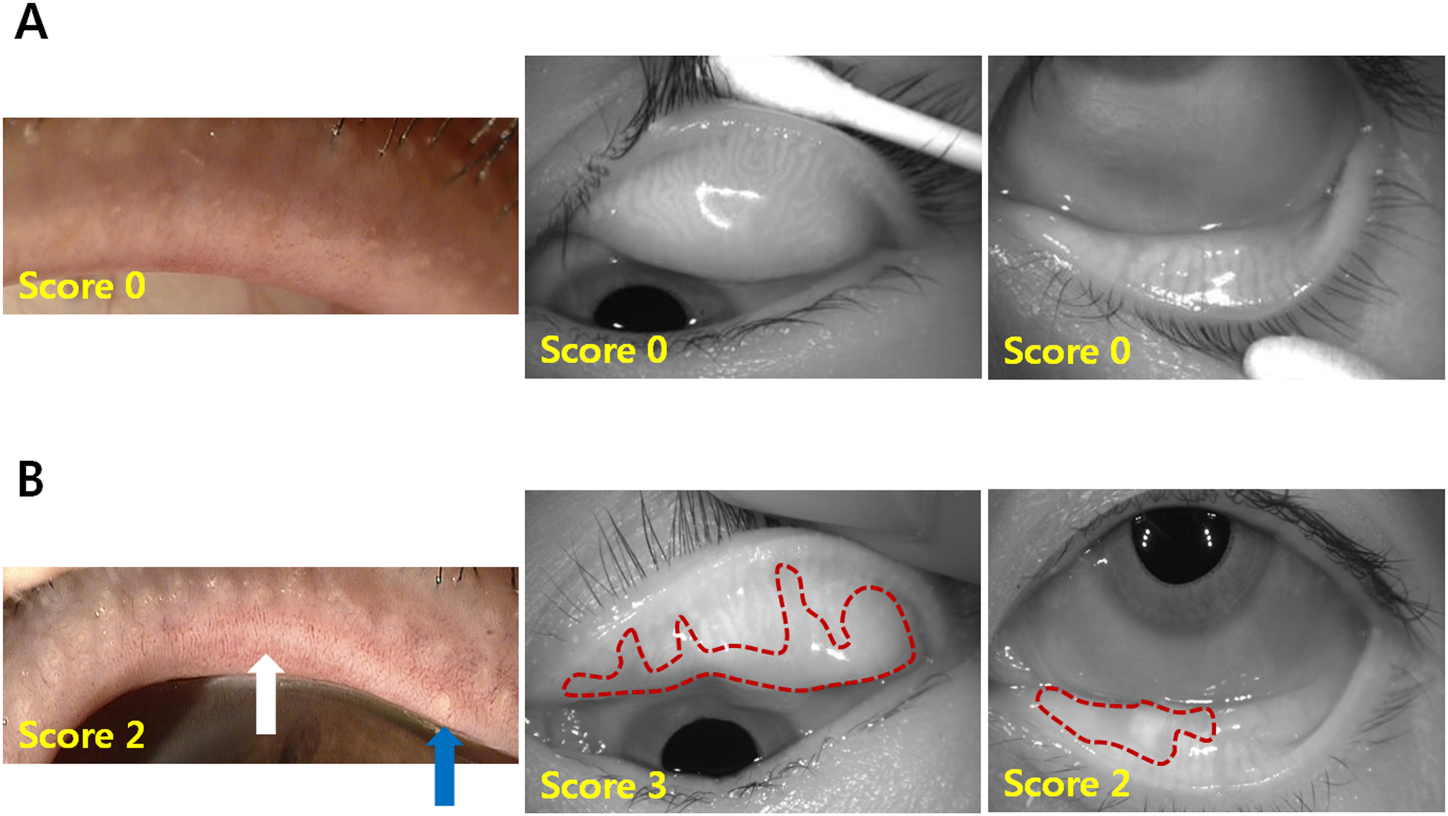
Representative images of lid margins and meibography of upper and lower lids in patients from control and NTG group. The score of each image is noted at the bottom left. (A) A 49-year-old man displays a normal eye in the control group: OSDI: 2.1; TBUT: 12 s; Schirmer’s test: 15mm; lid margin score: 0; meibum score: 0; and meibo score: 0 (B) A 52-year-old man displays meibomian gland dysfunction in the NTG group: OSDI: 22.92; TBUT: 3 s; Schirmer’s test: 7 mm; lid margin score: 2; meibum score: 1; meibo score: 5; and compliance score: 2.5. Vascular engorgement is outlined by the white arrow and a plugged meibomian gland orifice is outlined by the blue arrow. The areas of meibomian gland dropout are encircled with dotted red lines.

Multivariate analysis revealed that lid margin abnormality (OR, 0.256; 95% CI, 0.072–0.908), expression of meibum (OR, 0.144; 95% CI, 0.023–0.915), and meibomian gland dropout (OR, 0.344; 95% CI, 0.140–0.845) were significantly associated with medication compliance (Table 2). The representative images of meibomian gland parameters and medication compliance from typical patients of the control group and the NTG group are shown in Fig 1.

**Table 2:** Factors associated with medication compliance in the NTG group.

Variable	Univariate analysis		Multivariate analysis^a^	
	Odd ratio (95% CI)	P-value	Odds ratio (95% CI)	P-value
**Age (years)**	1.016 (0.976–1.058)	0.433		
**Sex (male)**	1.312 (0.373–4.616)	0.672		
**IOP (mmHg)**	0.828 (0.603–1.136)	0.242		
**MD (dB)**	1.020 (0.934–1.114)	0.659		
**PSD (dB)**	0.957 (0.820–1.117)	0.576		
**Duration of therapy (months)**	0.997 (0.979–1.015)	0.710		
**Type of PGAs**				
** Latanoprost**	reference			
** Tafluprost**	2.538 (0.548–11.766)	0.234		
** Bimatoprost**	1.385 (0.272–7.037)	0.695		
**Tear film parameters**				
** OSDI**	0.921 (0.822–1.032)	0.157		
** TBUT (sec)**	1.850 (1.052–3.255)	0.033	1.930 (0.871–4.273	0.105
** Schirmer’s test (mm)**	0.957 (0.672–1.364)	0.809		
** KEP**	0.723 (0.431–1.212)	0.218		
**Meibomian gland parameters**				
** Lid margin abnormality**	0.393 (0.185–0.834)	0.015	0.256 (0.072–0.908)	0.035
** Expression of meibum**	0.222 (0.076–0.650)	0.006	0.144 (0.023–0.915)	0.040
** Meibomian gland dropout**	0.405 (0.217–0.758)	0.005	0.593 (0.140–0.845)	0.020

NTG = normal tension glaucoma; IOP = intraocular pressure; MD = mean deviation; PSD = pattern standard deviation; PGAs = prostaglandin analogs; OSDI = ocular surface disease index; TBUT = tear film break-up time; KEP = keratoepitheliopathy

^a^ Only variables with a P value of less than 0.10 in the univariate analysis were included in the multivariate model.

**Fig 2:**
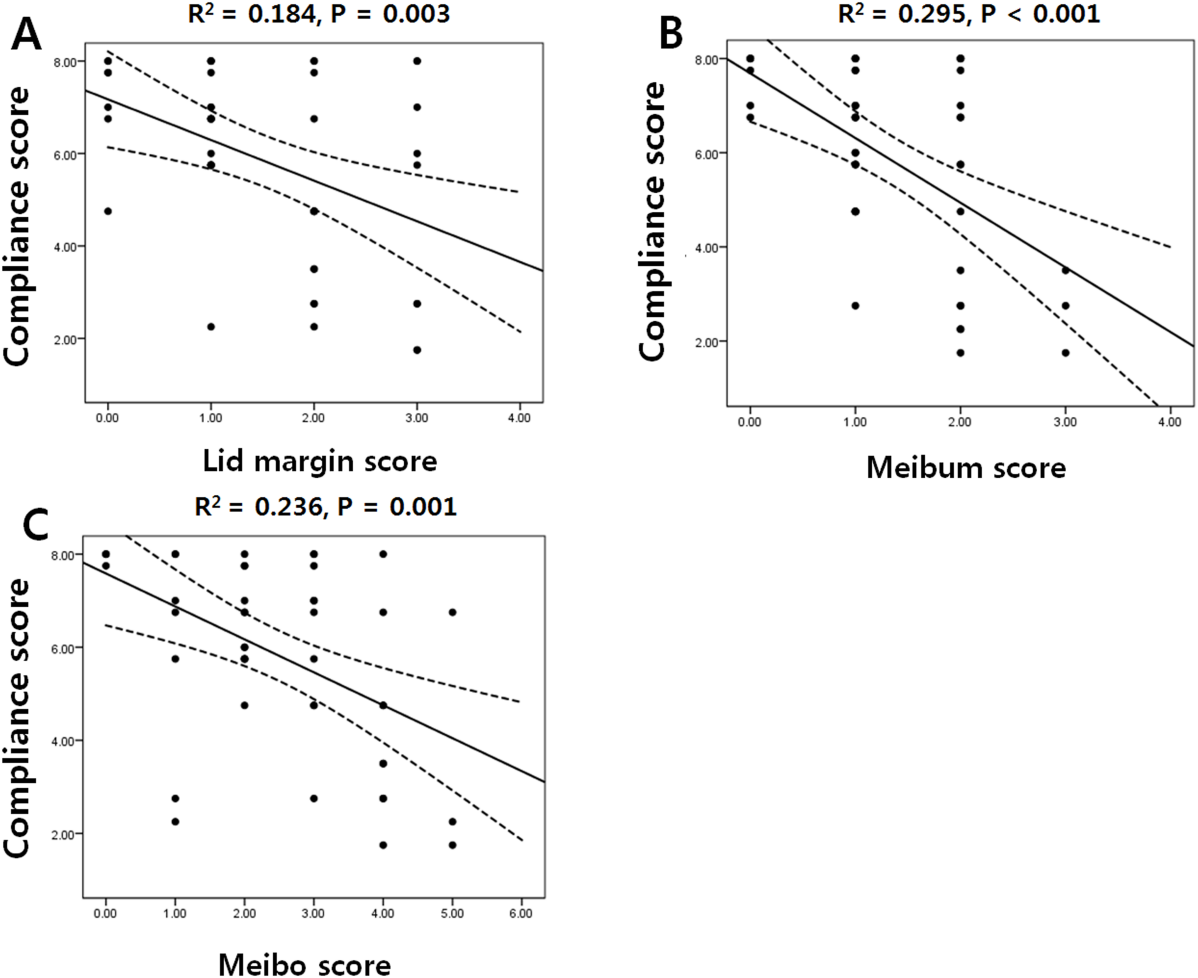
Scatter plots showing the relationship between meibomian gland parameters and medication compliance in the NTG group. (A) Lid margin score and compliance score, (B) Meibum score and compliance score, and (C) Meibo score and compliance score. The dashed lines represent the 95% confidence intervals for the solid trend lines.

Fig 2 shows the statistically significant association between the medication compliance and the score of meibomian gland parameters. Lid margin score (R^2^ = 0.184, P = 0.003), meibum score (R^2^ = 0.295, P < 0.001), and meibo score (R^2^ = 0.236, P = 0.001) showed a negative correlation with medication compliance.

## Discussion

Elevated IOP is a risk factor for the occurrence and progression of glaucoma. Therefore, lowering the IOP is vital for the monitoring and treatment of glaucoma [30–33]. Topical drugs used for lowering the IOP can be toxic to the ocular surface and reportedly increase the prevalence of OSD [34]. OSD related to topical anti-glaucoma drugs has been reported in many previous studies [3,5,14,34,35]. Furthermore, another study reported that the OSD in patients with glaucoma can influence the medication compliance [15]. However, no study has reported the relationship between the tear film and meibomian gland parameters and medication compliance in patients with glaucoma.

A previous study demonstrated that the topical anti-glaucoma medication group had a significantly shorter TBUT, greater fluorescein staining, and higher impression cytology grade compared to those of the control group [35]. Mocan et al. showed that the prevalence of MGD was higher in patients treated with PGA monotherapy than in patients no receiving PGAs [14]. The results of this study also showed that the group treated with topical PGAs had worse OSDI, shorter TBUT, greater corneal staining, and worse meibomian gland parameters compared to those of the control group. Other studies have reported that the OSD related to anti-glaucoma drugs was associated with the number of concomitant drugs and the number of instillations [36,37]. In this study, we selected patients with NTG treated with PGA monotherapy in order to control the number of drugs and instillations used. These inclusion criteria provide objective data for the tear film and meibomian gland parameters caused by medication in patients with glaucoma.

A previous study reported that a high prevalence of MGD was detected in patients who received PGA treatment [14]. Eyelid margin changes have been previously reported in association with PGAs in a report by Arita et al, in which 13 patients with glaucoma receiving PGA drops demonstrated higher lid margin change and meibography scores, and lower TBUT and Schirmer’s test score [13]. In this study, we assessed three parameters of MGD; lid margin abnormality score, meibum expression assessment, and the degree of meibomian gland dropout observed by Keratograph 5M. We also showed that the NTG group had significantly higher lid margin scores, meibum scores, and meibo scores compared with those of the control group. The mechanism involved in the meibomian gland changes induced by anti-glaucoma eye drops is unclear. Previous reports demonstrated that long-term therapy with multiple topical medications resulted in subclinical conjunctival inflammation [38,39]. Broadway et al. and Baudouin et al. demonstrated that anti-glaucoma eye drops increased the number of mast cells [40,41]. As mast cells mediate allergic responses, this increase may represent a shift toward a subclinical allergic reaction [40,42,43]. Chronic recurrent inflammation might also cause meibum stagnation, followed by keratinization of the orifices in the meibomian glands [44,45]. Possibly, prolonged exposure of the eyelid margin to topical PGA medications may induce keratinization of the meibomian gland acini, along with induction of hypertrichosis and periocular pigmentation, perhaps through a common molecular pathway [5]. In this study, all meibomian gland parameters were risk factors related to compliance, and were all worse in the NTG group than in the control group. Therefore, treatment of MGD may prove beneficial for patients on PGA monotherapy, who have not shown favorable compliance.

Meibomian gland function has been recognized as a critical factor in maintaining the ocular surface health and stability [46]. In this study, MGD parameters were the only factor associated with compliance. MGD has been defined as a chronic, diffuse abnormality of the meibomian glands, commonly characterized by terminal duct gland obstruction and/or qualitative/quantitative changes in glandular secretion [47]. This may result in alteration of the tear film, symptoms of eye irritation, clinically apparent inflammation, and ocular surface disease [28,48,49]. Furthermore, previous study reported that meibomian gland dropout was most common cause of evaporative dry eye [48]. In summary, MGD can cause tear film alteration, change of symptoms, inflammation, and ocular surface disease including evaporative dry eye. As a result, changes in meibomian gland, which is thought to be the critical cause of them, may be the only significant factor associated with compliance in patients with NTG.

Considering the previous report showing the dose response relationship between the duration and number of anti-glaucoma medications and the degree of the tear film and meibomian gland changes, one might question our results [50]. Better compliance means more frequent instillation of the PGA. We reasoned that the patient’s use of more frequent instillations of PGA causes higher scores of the meibomian gland parameters. However, our results revealed that medication compliance had a negative correlation with lid margin score, meibum score, and meibo score. Previous study reported that there were no significant differences in prevalence and severity of MGD based on the number of anti-glaucoma medications [14]. Another study demonstrated that the duration of anti-glaucoma treatment was not associated with tear film parameters [35]. Arita also revealed that there was no significant correlation between the meiboscore and the duration of therapy [13]. Therefore, the changes of the meibomian gland might not depend on the frequency of instillations or duration of therapy. These results suggest that the chronic use of PGAs can cause significant changes in the meibomian gland, if the patients experience changes beyond the threshold value caused by the long-term effect of medication.

The OSDI is a representative index of ocular discomfort. However, the OSDI was not a risk factor related to treatment compliance in this study. A previous study reported that even patients without ocular discomfort might develop signs of tear film instability and corneal epithelial damage [51]. Therefore, the OSDI may be not a representative factor associated with the tear film parameters, including MGD.

All patients in this study used BAK-preserved PGAs. Travoprost (Travatan^®^, Alcon Laboratories, Inc., Fort Worth, TX, USA) is also one of the representative PGA, but it is excluded from this study because it contains polyquad rather than BAK as a preservative. BAK has several positive attributes; however, it can also have dose-dependent detrimental effects on healthy ocular tissues. A BAK concentration of 0.0001% causes the arrest of cellular growth [52]. A concentration of 0.01% induces cellular apoptosis, and a concentration of 0.05–0.1% causes necrosis [53]. Therefore, the inherent detergent properties of BAK disrupt the lipid layer of the tear film, resulting in increased aqueous tear evaporation and decreased TBUT [51]. Shorter TBUTs related to PGAs in the NTG group might be attributable to the preservatives used in this study. However, the TBUT in patients with glaucoma was not a risk factor related to medication compliance in the multivariate analysis. Meibomian gland parameters were the only risk factors related to compliance. As a shorter TBUT might be due to MGD, it is not a causative factor. In this regard, comparative studies on the MGD due to the use of preservative-free PGA and preservative-containing PGA will be necessary in the future.

This study had several limitations. First, the sample size was small. Second, the study was performed using data from the same ethnic group; thus, results may not be applicable to other ethnic groups. Another limitation is that we only investigated associations with a single class of medication (PGAs). We did not analyze the relationship of other classes of medication, such as beta-blockers, carbonic anhydrase inhibitors, and α-2 adrenergic agonists, with tear film changes, including MGD. Further studies will be needed to identify such associations. Furthermore, we did not consider other side effects (PGAs associated periorbitopathy, deepening of upper eyelid sulcus, and growth of eyelashes) that could affect the compliance of PGAs. Lastly, clinical ocular surface tests cannot replace the morphological changes of MGD. The use of tools such as laser scanning confocal microscopy, which can directly confirm the morphological changes of MGD, will be needed in the future.

In conclusion, the malfunction of the meibomian glands (abnormalities of lid margins, meibum expressibility and meibomian gland dropout degree) can be factors associated with compliance of PGA monotherapy in patients with NTG. MGD may adversely contribute to the medication compliance in medically treated patients with glaucoma, and thus, a careful inspection of the eyelid margins in these patients for signs of MGD is recommended.
